# Anti-Aging Physiological Roles of Aryl Hydrocarbon Receptor and Its Dietary Regulators

**DOI:** 10.3390/ijms22010374

**Published:** 2020-12-31

**Authors:** Eva Serna, Cristina Cespedes, Jose Vina

**Affiliations:** Freshage Research Group, Department of Physiology, University of Valencia, CIBERFES, INCLIVA, 46010 Valencia, Spain; crisces@alumni.uv.es (C.C.); jose.vina@uv.es (J.V.)

**Keywords:** aryl hydrocarbon receptor, aging, physiological function

## Abstract

The vast majority of the literature on the aryl hydrocarbon receptor is concerned with its functions in xenobiotic detoxification. However, in the course of evolution, this receptor had to have physiological (rather than toxicological) functions. Our aim was to review the aryl hydrocarbon receptor’s role in the physiological functions involved in aging. This study was performed by searching the MEDLINE and Google Academic databases. A total of 34 articles were selected that focused specifically on the aryl hydrocarbon receptor and aging, the aryl hydrocarbon receptor and physiological functions, and the combination of both. This receptor’s main physiological functions (mediated by the modulation of gene expression) were cell regeneration, the immune reaction, intestinal homeostasis, and cell proliferation. Furthermore, it was shown that the loss of this receptor led to premature aging. This process may be caused by the dysregulation of hematopoietic stem cells, loss of glucose and lipid homeostasis, increase in inflammation, and deterioration of the brain. We conclude that the aryl hydrocarbon receptor, apart from its well-established role in xenobiotic detoxication, plays an important role in physiological functions and in the aging process. Modulation of the signaling pathway of this receptor could be a therapeutic target of interest in aging.

## 1. Introduction: Overview of AhR in Toxicology

The aryl hydrocarbon receptor (AhR) is a member of the family of basic helix–loop–helix transcription factors and is well-known for its role in xenobiotic metabolism and detoxification [1]. Although xenobiotics are all exogenous components found inside an organism, the scientific community, in this context, usually refers to dioxins, a class of polycyclic and halogenated aromatic hydrocarbons, with an almost planar structure and with very similar physical and chemical properties to each other.

Dioxins are generally anthropogenic compounds of industrial origin that, due to their high chemical stability, are persistent environmental pollutants.

Furthermore, since they are lipophilic, they tend to accumulate in the adipose tissue of animals, and in this way, they are incorporated into the food chain. These chemical compounds threaten not only animal life and ecological balances, but also human health. Mechanisms that regulate toxicity induced by dioxins through AhR activation are not fully understood at the molecular level and are a broad and relevant field of health study.

This receptor is considered an orphan receptor as it has a promiscuous ligand-binding site. It is capable of binding to a wide range of structurally different chemical compounds. This gives it great diversity in its functions and makes it a molecular receptor with high pharmacological potential [2]. AhR activity can also be modulated by food and even by endogenous ligands in humans [1].

The AhR is a ligand-activated receptor. Fukunaga and coworkers annotated the protein structure [3]. This activation, through heterodimer binding AhR/AhR nuclear translocator (ARNT) to short promoter elements called “dioxin responsive elements” (DRE), induces the transcription of enzymes responsible for xenobiotic metabolism, both phase I (cytochromes P450, (CYP450)) and phase II (transferases). In addition, the fact that enzymes induced by the binding of xenobiotics to AhR have degrading activity against these molecules has led to the idea that this pathway represents an adaptive metabolic response, which protects the body from exposure to a specific class of environmental pollutants [3]. However, the activation of this pathway does not always offer protection. In many cases, the induction of these enzymes leads to a metabolic activation/inactivation of their substrates and can convert them into compounds that are more potent carcinogens with adverse pathophysiological effects [4].

The binding of AhR to any of its ligands, both exogenous and endogenous, normally causes the activation of this receptor and its downstream signaling cascade. Before binding with ligands, AhR is in its latent form in the cytosol, associated with a protein complex, which stabilizes it and prevents its degradation by the proteasome in the absence of ligand. This protein complex is formed by two molecular chaperones in response to heat stress (HSP90), a chaperonin p23 and a HBV X-associated protein 2 (XAP2) also known as protein 9 associated with AhR (ARA9) or protein of interaction to AhR (AIP) [5]. HSP90s maintain the AhR in the necessary conformation to expose the ligand-binding area and limit entry into the nucleus by blocking the nuclear localization signal (NLS) [6]. When the AhR is activated by the binding of a ligand, a change occurs in the cellular compartmentalization of the receptor, which rapidly accumulates in the nucleus (translocation), and XAP is released. Thus, the AhR, once in the nucleus, interacts with the ARNT protein and releases the HSP90/p23 complex to recycle it in the cytoplasm. The AhR/ARNT complex binds to response elements (DRE) in the promoter regions of target genes, where it recruits transcription cofactors and proteins that remodel chromatin, promoting the transcription of a multitude of genes.

When the agonist capable of activating AhR signaling disappears, AhR activity must be rapidly inactivated to maintain cellular homeostasis.

Mechanisms are known to terminate AhR signaling; both involve in its degradation through the 26S proteasome. For this purpose, AhR must be exported from the nucleus, ubiquitinated and degraded by the proteasome in the cytosol [7] or through the binding of AhR to its repressor AhRR, thus establishing a negative feedback inhibitor of AhR signaling [8].

Overall, since the biological activity of AhR is controlled by its association with proteins present in different cellular compartments, the control of its intracellular location may represent a mechanism for regulating its activity under physiological conditions, i.e., in the absence of interaction with xenobiotics [9].

The AhR arose much earlier in the evolutionary scale than the presence of environmental pollutants. Therefore, it must have a constitutive, physiological activity in an organism. Its constitutive expression is found in invertebrates and nearly all vertebrates, both aquatic and terrestrial, and in almost all cell types in mammals [10].

The highest expression levels of AhR mRNA are found in the placenta, heart, liver, lung, and pancreas, and the lowest are found in the kidney, brain, and skeletal muscle [9]. It is also present in multiple vascular beds and even in tumor cells. In addition to the differential expression of the receptor in each type of tissue, its regulation also depends on the stage of development of the organism [3].

We have performed a comprehensive literature review to underpin the role of AhR under physiological conditions independently of its response to toxins. Our aim was to bring together our current knowledge on the physiological functions of AhR and the changes that occur during aging.

## 2. Physiological Functions of AhR

The oldest physiological function of the AhR in terms of evolution is the regulation of developmental processes observed in *Caenorhabditis elegans* and *Drosophila* [11].

The *C. elegans ahr* mutant develops slightly slower than the wild type [12] and shows a neuronal dysfunctional development [13,14,15,16]. *Drosophila*’s AhR homolog is necessary for development of the distal segments of the antennae [17] and sensory neurons [18].

Another conserved function is the regulation of fertility described in *C. elegans* and mice [12,19].

The importance of AhR in healthy development and in normal biochemical processes has been elucidated in studies using AhR gene knockout (KO) mice, which show numerous developmental abnormalities and dysfunctionalities [6]. However, other physiological functions require further investigation.

In 2014, Stockinger and his colleagues proposed that “the emphasis is shifting from AhR in the xenobiotic pathway to its mechanism action in response to its physiological ligands” [20].

Although the functions of the different endogenous ligands of the receptor are well characterized, none of these ligands acts as a high-affinity physiological activator. Further, there are differences in AhR activity and ligand reactivity between species. For instance, there are discrepancies in the experimental results between humans and mice [6]. In any case, AhR is involved in physiological processes such as embryogenesis, development, neurogenesis, circadian rhythm, metabolism, and hypoxia [21]. Furthermore, it also has a role in the proteasomal degradation of steroid hormone receptors, in the cellular response to stress caused by UVB radiation, and in the differentiation of specific T cell subtypes [22]. In Table 1, we summarize the functions known in which the receptor participates.

**Figure 1:**
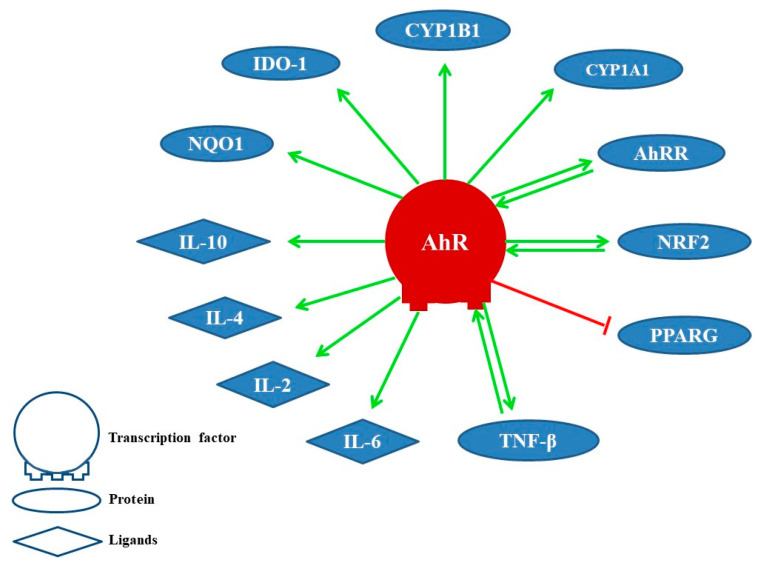
Proteins and ligands modulated by the AhR. Positive effect (link in green color) and negative effect (link in red color).

The activation of AhR induces cytochrome P450 enzymes, mainly CYP1A1 and CYP1B1, that detoxify xenobiotics and control the AhR signaling due to its ability to metabolize ligands. Therefore, it is presumed that metabolic clearance mediated by these enzymes for natural AhR ligands would affect various cell types that depend on AhR signaling for their survival [38,39]. In Figure 1, we show some proteins and ligands whose activity is modulated by AhR. Among them are AhR’s own repressor AhRR [31], the transcription factor related to nuclear erythroid factor 2 (NRF2, alias NFE2L2) [40,41], the gamma activated receptor for peroxisome proliferation (PPARG) [33,34], the transforming growth factor-beta (TGF-β) [42], interleukins (IL6, IL2, IL4, IL10) [43], NAD(P)H dehydrogenase (quinone) 1 (NQO1) [44], and indolamine 2,3-dioxygenase (IDO1) [20], among others.

Physiological AhR activity can be changed by endogenous ligands and nutrition [1]. AhR is reported as a crucial regulator in maintenance of intra-epithelial lymphocytes and microbial load composition in the intestine [45]. For this reason, AhR activity can be regulated by dietary components to improve the intestinal immune system and to avoid dysbiosis.

**Figure 2:**
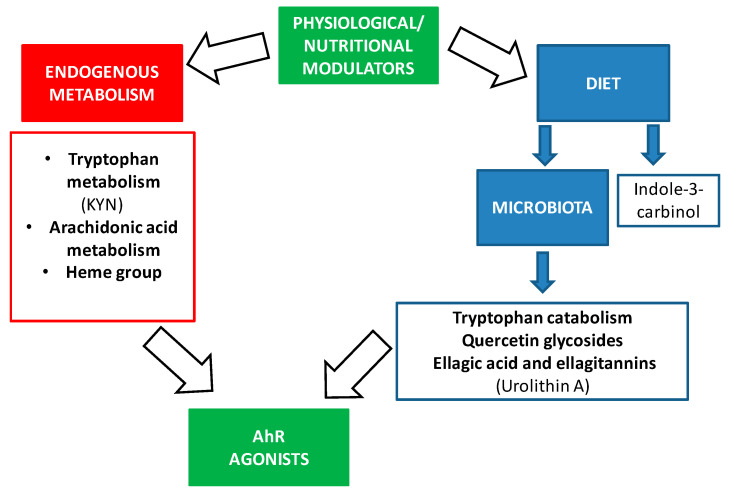
Physiological and nutritional modulators of AhR.

We summarize relevant modulators by AhR in Figure 2.

The most prominent endogenous ligands are those derived from tryptophan metabolism, but there are also ligands derived from arachidonic acid or the heme group (Figure 2). Of the catabolites derived from tryptophan, we would like to highlight the formation of n-formylquinurenine (KYN) by the enzymes indoleamine 2,3-dioxygenase (IDO) and tryptophan 2,3-dioxygenase (TDO) [46,47,48,49]. KYN is an AhR ligand that has been linked to the maturation of regulatory T cells and the suppression of inflammatory cytokines in dendritic cells. It also has a role in the response to microbial pathogens since lipopolysaccharides (LPS) stimulate the expression of IDO and TDO, leading KYN synthesis that in turn activates AhR. Thus, AhR decreases the expression of pro-inflammatory cytokines and regulates inflammation. Furthermore, in AhR gene KO mice, LPS induces higher concentrations of inflammatory cytokines and has a greater susceptibility to septic shock [50,51]. Under normal conditions, when LPS activates the Toll-like receptor, the enzyme TDO is expressed, leading to the formation of KYN that activates AhR in immune cells [52].

In addition, there are ligands for AhR that come from bacterial metabolism through the microbiota (Figure 2) [24]. AhR is highly expressed in intestinal epithelium. AhR expression was found to decrease in germ-free mice, suggesting that it is related to the microbiota [47]. The bacterial tryptophan catabolites that act as AhR ligands are indole, tryptamine, indoleethanol (IE), indolepropionic acid (IPA), indolelactic acid (ILA), indoleacetic acid (IAA), 3-methylindole (skatole), indolealdehyde (IAld), and indoleacrylic acid (IA) [48,53,54] (Figure 2). Thus, AhR mediates the regulation of intestinal immunity through both endogenous and bacterial tryptophan metabolites, having effects on the composition of the microbiota, the immune system, and intestinal homeostasis [24]. One of the mechanisms by which these metabolites contribute to the maintenance of intestinal homeostasis is the production of IL-22 by type 3 innate lymphoid cells (ILC), and this is also mediated by the activation of AhR [38,42,43,44,45]. IL-22 is important for the protection of the epithelium against pathogens and promotes the expansion of intestinal stem cells, promoting the regeneration of the epithelium after intestinal injury [55].

On the other hand, these bacterial tryptophan catabolites not only have consequences at the intestinal level, but they can be absorbed at the systemic level and have anti-inflammatory effects because, as mentioned above, the activation of AhR modifies the responses regulated by the Toll-Like receptor in dendritic cells and promotes the generation of T-helper cells that secrete IL-10 (anti-inflammatory) [56]. It should be noted that these studies are conducted in mice, and the results may differ in humans since the affinities of metabolites for AhR may be different. Recent studies suggest that the human AhR has a greater affinity for tryptophan catabolites than that of the mouse [54]. In addition, the diet can provide indole derivatives, like indole-3-carbinol (I3C), that directly activate the AhR signaling pathway [57].

An improved healthspan effect in *C. elegans*, *Drosophila melanogaster*, and mice has also been attributed to indoles [58].

Other dietary components can also be metabolized by the microbiota giving rise to AhR agonists as shown in Table 2.

Urolithin A (UroA) is a derivative of elaginates; the successive hydrolysis and decarboxylation of elaginates by the intestinal microbiota form urolithins. Interindividual differences in urolithin formation depend on the composition of the microflora [63]. UroA shows anti-cancer, anti-inflammatory, and anti-aging effects. This metabolite has the ability to reduce the production of IL-6 and TNF by macrophages and to bind to AhR in intestinal cells.

## 3. The Implication of AhR in Aging

Population aging is a global demographic trend. The World Health Organization estimates that the proportion of the world population ≥65 years will reach 1500 million people (14% of the total) by 2050. Age-related changes contribute to the appearance of diseases that occur more frequently with aging [64]. This process significantly increases the prevalence of chronic age-related conditions such as heart and neurological diseases, cognitive decline, and dementia.

There is an interesting association between the AhR expression levels and longevity [27].

A pioneer study by Fernández-Salguero and his collaborators confirmed that AhR is affected in aging, leading to a deterioration of several physiological functions [21]. Thus, the detailed characterization of AhR functions in aging and age-related diseases could help discover this protein’s ancient functions and develop strategies for healthier aging. In this study, the role of AhR in the aging process was analyzed using a model *Ahr* gene KO mice. Almost 50% of the mice died or fell ill at 13 months of age (average half-life of mice is approximately 24 months). The most notorious changes were cardiovascular diseases (hypertrophy, focal inflammation, hypertrophic vessels, and vascular lesions), liver (portal vascular hypertrophy and hepatocellular tumors), hyperplasia gastric with aging to polyps, the immune system with deficiency of T and B cells, rectal prolapse, and severe epidermal hyperplasia. Age-related lesions in various organs appeared only at nine months of age, for instance, alterations in cell proliferation, fibrosis, collapse of the immune system, and alterations in vascular homeostasis. None of these complications was found in wild-type (WT) mice of similar ages [21]. Bravo-Ferrer and coworkers corroborated the accelerated aging phenotype in *Ahr*-deficient mice [43].

Singh and collaborators showed implications of the AhR in regulating the HSC [65]. Normally, HSCs are largely inactive in order to prevent premature exhaustion and limit genetic alterations. Singh et al. showed that the HSC of young adult *Ahr* KO mice had high rates of cell division. These KO mice showed higher numbers of bone marrow cells. A similar survival rate was also observed in WT mice until approximately 15 months of age. At 24 months, only 33% of deficient mice survived in comparison with 78% of WT mice. Therefore, the lack of expression of *Ahr* allows HSCs to escape inactivity and lose control over the signals that limit proliferation and differentiation, ultimately resulting in stem cell exhaustion and premature aging.

Biljes and collaborators carried out a study in young (from 2 to 5 months) and old (>18 months) mice, with or without *Ahr* gene deficiency [22]. In young KO mice, they found that postprandial TG, cholesterol, VLDL, and HDL levels were significantly lower than in the WT ones. LDL levels were not changed. Unexpectedly, there was no difference in any of these lipid markers between *Ahr* KO old and young; therefore, there were no age-associated changes in KO mice concerning these parameters. However, expression of lipoprotein lipase (LpL), which is the enzyme that degrades the lipid moiety of lipoproteins and therefore releases TG, increased with age in the livers of WT and KO mice, but it was higher in deficient mice than in wild-type mice. The LpL has an assumed DRE transcription motif in its promoter and therefore might need the AhR for constitutive expression. This could explain why the low TG levels in LpL KO mice are very similar to the human situation, making the mouse a relevant model for the study of lipid metabolism [22].

The prevalence of type 2 diabetes increases with age. In studies to evaluate the potential role of AhR in altering the metabolism of glucose, authors observed that in the KO mice the blood glucose levels were significantly higher in the old mice (>18 months) than in young (from 2 to 5 months), indicating that old *Ahr* KO mice cannot maintain blood glucose homeostasis [23].

At the vascular aging level, Eckers et al. observed that mice deficient in the *Ahr* gene showed a decrease in pulse wave velocity in young and old animals compared to wild types. This indicates a reduced stiffness of the arteries and, therefore, healthier vessels. However, overexpression of *AhR* alters eNOS activation and reduces S-NO content in human endothelial cells; moreover, pulse wave velocity is positively correlated with *AhR* expression in healthy human subjects [9].

The study by Bravo-Ferrer and collaborators revealed the participation of AhR in inflammation in aging. Its dysregulation could contribute to the loss of homeostasis that occurs during aging. The inflammation markers profile was compared in WT and *Ahr* KO mice at 2, 12, and 16 months. The *Ahr* KO mice showed higher plasma levels of IL6, TNFα, IL2, IL4, and IL10 than WT mice, and lower in IL6, IFNγ, IL17α, IL2, and IL4 with age in KO mice compared to WT. In both groups of aged mice there were signs of inflammation, but KO mice showed levels of specific cytokines higher than WT. These results indicate that the lack of AhR promotes an age-related increase in plasma cytokine levels and further supports the hypothesis that the loss of AhR promotes premature aging in mice.

Age causes changes in AhR expression levels in the mouse brain [43]. Indeed, AhR levels fall in the cortex and the hippocampus, but this is not reflected in its transcriptional activity as determined by levels of its target genes, CYP1A1 and CYP1B1. Moreover, the size of specific brain structures as determined by magnetic resonance imaging in WT and KO in 16-month-old mice was reduced when compared with WT animals. The results showed that the KO mice had a cortical volume reduction compared to WT animals. They also showed that the KO mice had a loss of the white matter integrity compared to WT.

The discovery that AhR protein levels decrease during aging in specific brain structures suggests the role of the AhR in brain aging. Moreover, because brain aging is associated with cognitive impairment, researchers have also assessed cognitive function throughout aging. This age-associated loss of cognition is increased in *Ahr* KO mice. These early-aging characteristics were not present in WT or young KO mice. Furthermore, administration of a diet rich in xenobiotics, as commonly occurs in the aging population, accelerates the appearance of β-amyloid plaques in the mouse brain and deficits in working memory in aged mice [43].

The processes related to AhR dysregulation in aging are summarized in the following Table 3.

In addition to these processes, kynurenine (KYN) levels increase in aging [43]. These high levels are associated with more significant inflammation and seem to contribute directly to the development and progression of various age-related conditions. When KYN interacts with AhR, reactive oxygen species’ levels are increased, leading to loss of characteristic muscle mass (sarcopenia) in old age. Lamas and coworkers’ studies argue that the concentration of KYN required to trigger AhR activity casts doubt on its relevance as an AhR activator in physiological conditions [6]. Furthermore, IDO, which is the enzyme that metabolizes tryptophan giving rise to KYN, may represent a new approach therapy for the prevention of sarcopenia and possibly other age-associated conditions [65]. In *C. elegans*, indoles induce a transcriptomic profile in aged animals similar to that of young ones, but this is different from that associated with normal aging [58].

**Figure 3:**
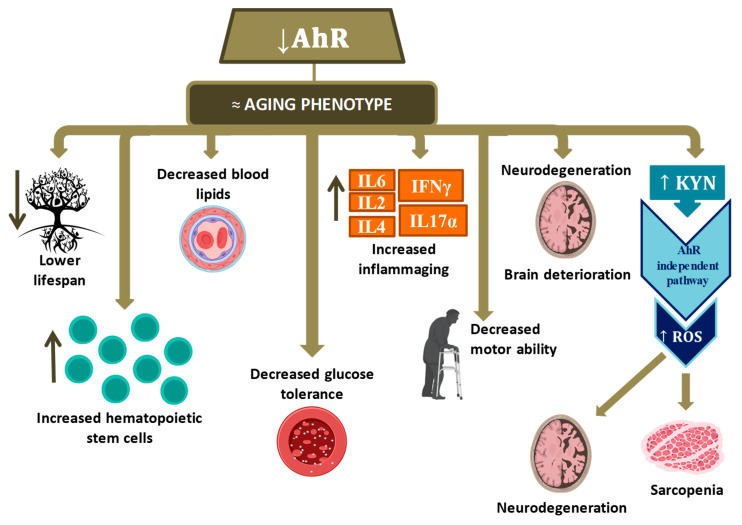
Pathophysiological processes observed when AhR decreases in aging. Hematopoietic stem cells (HSC); Interleukin (IL); Interferon (INF); Kynurenine (KYN); Reactive oxygen species (ROS). Up arrow means it increases and down arrow means it decreases.

The available literature data are summarized in Figure 3.

### Preliminary Results of AhR Signaling in Human Aging

Our research group performed a transcriptomic analysis of mononuclear cells in blood in young people, septuagenarians, and centenarians [65]. This is, to our knowledge, the first study in which AhR changes were analyzed in human longevity, including extreme longevity (centenarians), see Table 4. All materials and methods are described in detail in [66] and summarized in Supplementary Materials.

Table 4 shows that older people (ordinary aging) have the entire *AhR/ARNT* pathway underexpressed, including some of the key CYP450 genes (*CYP1B1*). The repressor of the pathway, *AhRR*, is increased. This result corroborates the importance of AhR during ordinary aging in humans.

**Figure 4:**
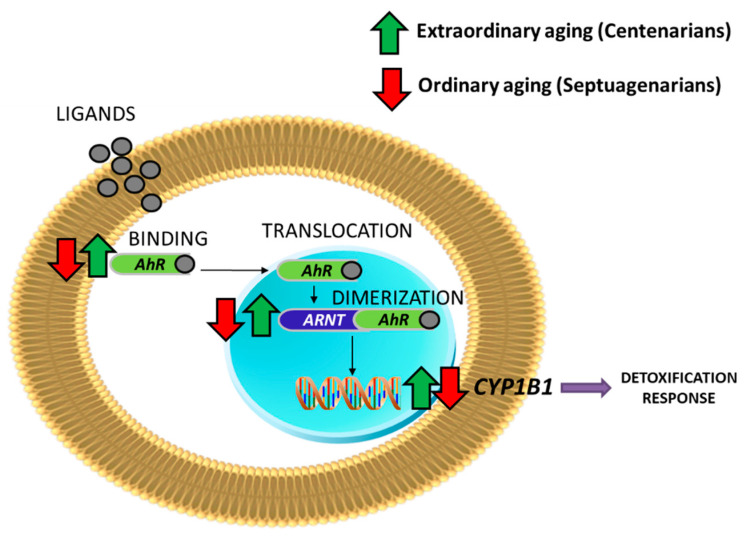
AhR signaling in centenarians is up-regulated in comparison with septuagenarians.

In the case of the centenarian population (extraordinary aging), we observed that they maintained the expression of the *AhR* pathway as high as that in the young, and when compared with the septuagenarians it was significantly overexpressed (Table 4 and Figure 4).

## 4. Conclusions

This review shows that AhR has a role in multiple physiological functions because it participates in processes such as gene expression, cell regeneration, immune response, intestinal homeostasis, skin pathogenesis, retinal homeostasis, and insulin regulation, among others. Because it mediates many physiological processes, it is considered a homeostatic sensor, and its dysregulation may favor the appearance of pathophysiological processes.

There is a decrease in AhR levels in aging, and this may be related to a series of phenotypic characteristics, such as proliferative dysregulation cell, loss of homeostasis in lipids and glucose metabolism, the appearance of an inflammatory state, decreasing motor capacity, and cerebral and vascular deterioration. Mice lacking this receptor display accelerated aging that produces more premature and severe aging phenotypes.

We have shown in humans that this receptor is notably up-regulated in centenarians, but these studies must be followed up to determine whether the observed changes are relevant to human aging.

The search for therapeutic targets of the AhR signaling pathway to modulate the physiological processes associated with age is warranted.

## 5. Materials and Methods

The literature review was performed by conducting electronic searches of Google Academic and MEDLINE. The electronic search used the following keywords and MeSH terms: (i) Aryl hydrocarbon receptor AND Aging; (ii) Aryl hydrocarbon receptor AND Physiological functions; (iii) Aryl hydrocarbon receptor AND Aging AND Physiological functions.

No publication date limits were set.

For a complete overview of the studies, the inclusion and exclusion criteria established to focus on the work’s objective are listed in Table 5.

**Figure 5:**
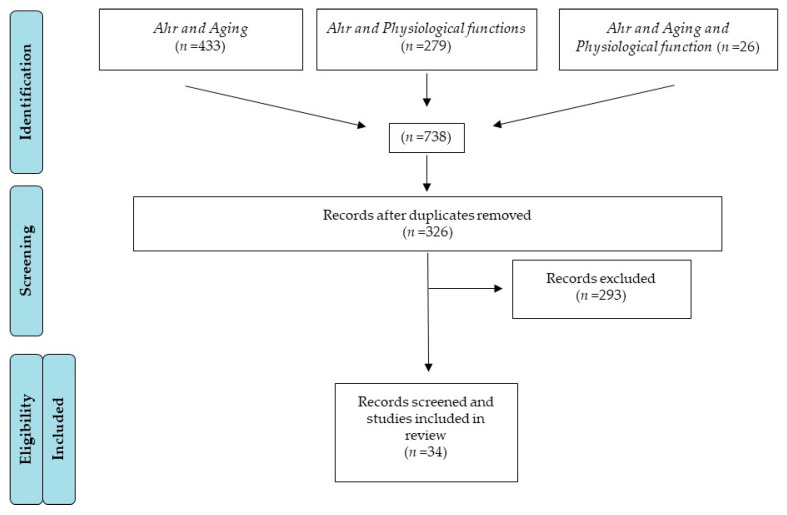
PRISMA flow diagram of literature review process for this study.

A total of 738 articles were found from a combination of the searches. After eliminating duplications, there were 326 articles, but only 34 fulfilled the inclusion criteria, as shown in the flow diagram below (Figure 5). References to AhR ligands, which are included in the text, are not considered in this scheme as this is restricted to “Aging” and “Physiological functions”.

## Figures and Tables

**Table 1:** The effect of aryl hydrocarbon receptor (AhR) signaling in physiological functions.

Function	Effect on the AhR Pathway	References
Cell regeneration	Activation	[23]
Immune reaction	Activation	[24,25,26]
Intestinal homeostasis	Activation	[6,25]
Hematopoietic stem cell (HSC) proliferation	Inhibition	[27]
Skin pathogenesis	Inhibition	[28]
Neurogenesis and neural precursor cells	Activation	[2,27]
Retinal homeostasis	Activation	[29]
Regulation of sex hormones and reproduction	Activation	[19,30,31]
Embryonic cardiac development	Activation	[32]
Insulin–glucose regulation	Activation	[23,32]
Adipocyte differentiation	Inhibition	[33,34]
Closure of ductus venous	Activation	[35]
Thickness aorta	Inhibition	[36]
Movement regulation	Inhibition	[37]

**Table 2:** Dietary components and microbiota metabolites that are AhR ligands.

Dietary Components	Microbiota Metabolites That Are AhR Ligands	References
Tryptophan	Indole, tryptamine, indoleethanol (IE), indolepropionic acid (IPA), indolelactic acid (ILA), indoleacetic acid (IAA), 3-methylindole (skatole), indolealdehyde (IAld), and indoleacrylic acid (IA)	[46,49,55]
	Indirubin	[59]
	1,4-Dihydroxy-2-naphthoic acid (DHNA)	[60]
Quercetin glycosides	3,4-Dihydroxyphenylacetic acid (DOPAC)	[61]
Ellagic acid and ellagitannins	Urolithin A	[62]

**Table 3:** AhR dysregulation in aging.

Processes	References
Dysregulation of hematopoietic stem cells (HSC)	[64]
Dysregulation of lipid metabolism	[22]
Loss of glucose homeostasis	[22]
Increased inflammaging	[43]
Impaired motor ability	[43]
Brain and vascular deterioration	[9,43]

**Table 4:** Differential expression genes involved in the AhR pathway.

	*AhR*	*ARNT*	*CYP1B1*	*AhRR*
Septuagenarians vs. Young people	−2.04 (*p* value = 0.004)	−1.62 (*p* value = 0.05)	−1.46 (*p* value = 0.04)	1.34 (*p* value = 0.01)
Centenarians vs. Septuagenarians	2.37 (*p* -value = 0.001)	2.41 (*p* value = 0.001)	1.87 (*p* value = 0.002)	−1.53 (*p* value = 0.0008)
Centenarians vs. Young people	1.16 (NC)	1.49 (NC)	1.20 (NC)	−1.13 (NC)

NC = no changes were observed. Negative numbers indicate underexpression compared to the corresponding controls; positive indicates overexpression.

**Table 5:** Inclusion and exclusion criteria.

Inclusion Criteria	Exclusion Criteria
Scientific articles in the English language	Scientific articles related to AhR in cancer or others pathologies
Full text available	Scientific articles related to AhR in the field of toxicology with exogenous ligands (xenobiotics)

## Data Availability

Raw microarray data will be available from ArrayExpress database (https://www.ebi.ac.uk/arrayexpress/).

## Supplementary Materials

The following are available online at https://www.mdpi.com/1422-0067/22/1/374/s1. METHODS. Study population. Peripheral blood mononuclear cells isolation. Isolation of total RNA from peripheral blood mononuclear cells. Gene expression profiling. Microarray data analysis.

## Abbreviations

AhR	Aryl Hydrocarbon Receptor
AhRR	Aryl-Hydrocarbon Receptor Repressor
ARNT	Aryl Hydrocarbon Receptor Nuclear Translocator
CYP450	Cytochromes P450
DRE	Dioxin responsive elements
HDL	High-Density Lipoprotein
HSC	Hematopoietic Stem Cells
IDO	Indoleamine 2,3-DiOxygenase
KO	Knockout
KYN	Kynurenine
LDL	Lipoprotein of Low Density
LpL	Lipoprotein Lipase
LPS	Lipopolysaccharides
ROS	Reactive Oxygen Species
TCDD	2,3,7,8-TetraChloroDibenzo-p-Dioxin
TDO	Tryptophan 2,3-DiOxygenase
TG	Triglycerides
UroA	Urolithin A
VLDL	Very Low-Density Lipoprotein
