# Supplementary material for: Anti-Aging Physiological Roles of Aryl Hydrocarbon Receptor and Its Dietary Regulators
Source: Int J Mol Sci. 2020 Dec 31;22(1):374. doi: 10.3390/ijms22010374 (PMC7795126; doi:10.3390/ijms22010374)
Supplement: Supplementary file 1 [file ijms-22-00374-s001.pdf]

METHODS

Study population

The Spanish Centenarian Study Group at RETICEF began in 2007 as a population-based study of all centenarians living within an area near of Valencia called La Ribera (11th Health Department of the Valencian Community, Spain), which comprises 29 towns (240,000 inhabitants). Potential subjects were selected from the population data system of the 11th Health Department. We found 31 centenarians of whom 20 met the inclusion criteria. Then, we randomly recruited 20 septuagenarians of whom 16 met the inclusion criteria and 20 young people of whom 14 fulfilled the inclusion criteria. The inclusion criteria were: to be born within the dates indicated in the study (before 1918 for centenarians, between 1928 and 1938 for septuagenarians, and between 1968 and 1988 for young individuals); to live in the 11th Health Department for the past ≥6 years; and to provide informed consent. The sole exclusion criterion was to be terminally ill for any reason. All experimental procedures were approved by the Committee for Ethics in Clinical Research of the Hospital de la Ribera, Alzira. All patients or their relatives were fully informed of the aims and scope of the research and signed an informed consent.

Peripheral blood mononuclear cells isolation

Whole blood collected in one VACUTAINER® CPT™ (BD, Franklin Lakes, NJ) containing sodium heparin as anticoagulant was collected from each subject. Within 0.5 hours of collection, blood was processed at the collection site according to the manufacturer's instructions by centrifugation at 3000 × g at room temperature for 15 minutes. After centrifugation, the cell preparation tubes were gently inverted several times to separate plasma, mononuclear cells, and erythrocytes. We collected the white ring containing mononuclear cells. Mononuclear cells were washed twice in PBS and frozen at –80ºC for subsequent RNA isolation.

Isolation of total RNA from peripheral blood mononuclear cells

Total RNA was isolated using a mirVana miRNA Isolation Kit (Ambion, Austin, TX) according to the manufacturer's directions. The purity and concentration of RNA were determined from OD260/280 readings by Genequant Pro Classic spectrophotometer (GE Healthcare). RNA integrity was determined by capillary electrophoresis using the RNA 6000 Nano Lab-on-a-Chip kit and the Bioanalyzer 2100 (Agilent Technologies, Santa Clara, CA). Only RNA extracts with RNA integrity number of ≥6 underwent further analysis.

Gene expression profiling

mRNA profiling was performed using GeneChip Human Gene 1.0ST Array (Affymetrix, Santa Clara, CA, USA). This array comprises>750,000 unique 25- mer oligonucleotide transcripts constituting over 28,000 well-annotated genes.

Microarray experiments were conducted according to the manufacturer's instructions. Briefly, 200 ng total RNA was labeled using WT Expression Kit (Ambion). The labeling reaction was hybridized on the Human Gene Array in Hybridization Oven 640 (Affymetrix) at 45°C for 18 hours. The arrays for each group were stained with Fluidics Station 450 using fluidics script FS450_0007 (Affymetrix) then scanned on GeneChip Scanner 3000 7G (Affymetrix). GeneChip® Command Console®software supplied by Affymetrix was used to perform gene expression analysis.

Microarray data analysis

Data (. CEL files) were analyzed and statistically filtered using software Partek Genomic Suite 6.4 (Partek Inc., St. Louis, MO). Input files were normalized with the RMA algorithm for gene array on core metaprobesets. A 1-way ANOVA was performed with the Partek Genomics Suite across all samples. Statistically significant genes between different groups were identified using a model analysis of variance with P value ≤0.05. The imported data were analyzed by Principal Components Analysis to determine the significant sources of variability in the data. Finally, the selected genes, specified for centenarian group, were imported into Pathway Studio v8 (Ariadne software) to classify the molecular function and biological processes represented by the mRNAs differentially expressed in the intersection between centenarians versus young and centenarians versus normal aging.
